# Factors associated with exclusive breastfeeding in Israel during the COVID-19 pandemic: a subset of the IMAgiNE EURO cross-sectional study

**DOI:** 10.1186/s13006-023-00568-y

**Published:** 2023-06-09

**Authors:** Rada Artzi-Medvedik, Ilaria Mariani, Emanuelle Pessa Valente, Marzia Lazzerini, Ilana Azulay Chertok

**Affiliations:** 1 School of Nursing, College of Health Sciences and Professions, Ohio University, Athens, OH USA; 2 WHO Collaborating Centre for Maternal and Child Health, Institute for Maternal and Child Health IRCCS Burlo Garofolo, Trieste, Italy; 3 Maternal Adolescent Reproductive and Child Health Care Centre, London School of Hygiene & Tropical Medicine, London, UK

**Keywords:** Exclusive breastfeeding, Breastfeeding at discharge, COVID-19 pandemic, Maternity care indicators

## Abstract

**Background:**

Evidence has shown that restrictions during the COVID-19 pandemic have negatively affected breastfeeding support and outcomes in hospitals in many countries. The aims of the study were to describe exclusive breastfeeding rates and identify factors associated with exclusive breastfeeding at hospital discharge among women who gave birth during the COVID-19 pandemic in Israel.

**Methods:**

A cross-sectional online anonymous survey based on WHO standards for improving quality of maternal and newborn care in health facilities was conducted among a sample of women who gave birth to a healthy singleton infant in Israel during the pandemic (between March 2020 and April 2022). The socio-ecological approach was employed to examine intrapersonal, interpersonal, organizational, and community/society factors associated with exclusive breastfeeding at hospital discharge according to women perspectives.

**Results:**

Among the 235 Israeli participants, 68.1% exclusively breastfed, 27.7% partially breastfed, and 4.2% did not breastfeed at discharge. Results of the adjusted logistic regression model showed that factors significantly associated with exclusive breastfeeding were the intrapersonal factor of multiparity (adjusted OR 2.09; 95% Confidence Interval 1.01,4.35) and the organizational factors of early breastfeeding in the first hour (aOR 2.17; 95% CI 1.06,4.45), and rooming-in (aOR 2.68; 95% CI 1.41,5.07).

**Conclusions:**

Facilitating early breastfeeding initiation and supporting rooming-in are critical to promoting exclusive breastfeeding. These factors, reflecting hospital policies and practices, along with parity, are significantly associated with breastfeeding outcomes and highlight the influential role of the maternity environment during the COVID-19 pandemic. Maternity care in hospitals should follow evidence-based breastfeeding recommendations also during the pandemic, promoting early exclusive breastfeeding and rooming-in among all women, with particular attention to providing lactation support to primiparous women.

**Trial registration:**

Clinical Trials NCT04847336.

## Background

Exclusive breastfeeding affords infants immunoprotection, supports infant growth and development [1], reduces the risk of respiratory and gastrointestinal infections [2], and was associated with lower mortality rates than infant formula feeding [3]. In addition, bacteria in human milk contribute to the development of the infant gut microbiome [4] and the intestinal barrier, thereby enhancing the infant’s immunologic maturation [5]. During the COVID-19 pandemic, exclusive breastfeeding was critical in protecting infants from COVID-19 infection as researchers found that human milk contains virus specific IgA and IgG antibodies following immunization [6].

Regarding pre-pandemic early breastfeeding rates in Israel, according to the Israel Ministry of Health (MOH) 2019–2020 preliminary report, in a random sample of 1,502 surveyed mothers, the overall breastfeeding initiation rates were 92.0%, although the majority of infants (80.1%) were fed infant formula at least once in the hospital prior to discharge (without medical indication) [7]. Examining breastfeeding rates in 2015–2017 in central Israel, typically defined as Tel Aviv/Jaffa and the surrounding area, researchers found significant differences in the rates and factors associated with exclusive breastfeeding at hospital discharge according to ethnic-cultural background [8]. In another study, overall breastfeeding initiation rates post-discharge were 92.2% among 868 women attending maternal child health clinics (*Tipat Halav*) in the Jerusalem district in 2017–2018, with significant differences based on ethno-cultural and religious background [9].

Israel has experienced a reduction in lactation support and in exclusive breastfeeding rates during the pandemic [10], as has been found in many countries [11–14]. Among 580 Israeli mothers surveyed early in the pandemic (April to May 2020), 127 (22%) women reported changes in their lactation intention: 85 (15%) responded that they extended their breastfeeding during the lockdown period and 42 (7%) reported shortening their intended duration. More than 90% of these respondents believed that breastfeeding counselling in the hospital and post-discharge in the community would facilitate breastfeeding [10]. Additional challenges included maternal-infant separation during early postpartum and decreased skin-to-skin contact, which are associated with low rates of exclusive breastfeeding [15]. Similar practices that impeded exclusive breastfeeding were found among maternity facilities in large multi-country studies [11, 13]. Furthermore, the lack of consistent, evidence-based guidelines aligned with the revised breastfeeding guidelines that were published by the World Health Organization (WHO) in June 2020 [16] compounded the challenges to exclusive breastfeeding in hospitals [13].

The current study is part of the IMAgiNE EURO project, a large multi-country survey conducted in more than 20 countries in the WHO European Region to collect views of women on the quality of maternal and newborn healthcare during and beyond the COVID-19 pandemic [17]. The aim of the current study was to identify factors associated with exclusive breastfeeding at discharge, a quality measure of facility-based maternity care [18], among women who gave birth during the COVID-19 in Israel.

## Methods

The study followed the General Data Protection Regulation and was registered in Clinical Trials (ref. NCT04847336). The protocol was approved by the coordinating center’s institutional review board and then reviewed and approved or deemed exempt by the ethical committees of other participating researchers’ countries. Detailed description of the survey and its development and validation has been previously reported [19]. Overall, the survey included 40 Quality Measures based on WHO Standards for Improving Quality of Maternal and Newborn Care in Health Facilities [20] which included breastfeeding practices indicators. The socio-ecological approach was used to examine the intrapersonal, interpersonal, organizational, and community and society factors associated with exclusive breastfeeding in a comprehensive manner, as has been applied in previous breastfeeding research [13, 21].

Mothers at least 18 years old who gave birth to a singleton, live infant in a facility in Israel during the COVID-19 pandemic outbreak (between March 2020 and July 2022) were eligible to participate in the survey, whereas home births, stillbirths, twin or multiple births, infants admitted to the neonatal intensive care unit (NICU) or special care baby unit, and mothers admitted to the intensive care unit (ICU) were excluded from the analysis. Additionally, mothers with more than 20% missing data on the Quality Measures variables and five key socio-demographic variables: date of birth, maternal age, education, parity, and immigrant status were excluded from the analysis.

Mothers was recruited through social media and through lactation forums and networks, to increase accessibility to breastfeeding women. Informed consent was obtained prior to participation through the online survey link. Participants in Israel had the option of choosing to answer the anonymous survey in Hebrew, English, or any of the 23 languages available during the study period. A total of 316 women who gave birth in Israel during the COVID-19 pandemic completed the survey and met inclusion criteria, of whom 235 had less than 20% missing data and were included in the analysis.

### Data analysis

The primary outcome of this study was exclusive breastfeeding at discharge. The specific question asked to mothers during the online survey was: “How were you feeding your baby when you were discharged from the hospital?” Mothers answers were defined as the dichotomous variable of exclusive breastfeeding versus partial or no breastfeeding (non-exclusive breastfeeding, where maternal choice of formula use is included in this option). Based on the outcome of exclusive breastfeeding, a sample of 203 mothers was needed to estimate exclusive breastfeeding based on an expected rate of 75% ± 5% with a 90% confidence interval [22].

Relevant variables to exclusive breastfeeding were included in the analysis based on previous breastfeeding research that applied the socio-ecological model [21]. The independent variables were categorized according to the domains of the socio-ecological model. Intrapersonal factors included the sociodemographic factors of maternal age, parity, education, and giving birth in a different country than mother’s origin (indicating immigrant status), and perinatal factors during the COVID-19 pandemic including difficulty attending routine prenatal care, faced barriers to prenatal care (including logistic, financial, lockdown, and lack of childcare), and mode of birth. Interpersonal factors related to social support of the mother are represented by adequate visiting hours for partner or relatives and presence of companion of choice. Organizational factors included skin-to-skin contact in the first hour, early breastfeeding initiation in the first hour, rooming-in (including night), perceived adequacy of breastfeeding support, and perceived professionalism of healthcare providers. For the community and society factors variables included were geographic region of birth in Israel (central, Jerusalem district, north, and south), the timing of 50% COVID-19 vaccination of the population [23], and the timing of the variant outbreaks in Israel (SARS-CoV-2 alpha, delta, and omicron) [24].

Descriptive analysis was conducted to calculate frequencies and proportions. Chi-square tests and Fisher’s exact tests were used to compare differences in exclusive breastfeeding for each independent variable. For the primary study aim, multivariable logistic regression was employed to identify the variables significantly associated with exclusive breastfeeding, using a backward stepwise variable selection to identify independent variables to be included in the final model. Adjusted odds ratio (aOR) and 95% confidence intervals (CI) were reported for each independent variable, with adjustment for maternal age, mode of birth, and giving birth in same country as mother’s origin. Additionally, for mothers who reported their COVID-19 status (a question that was not mandatory for questionnaire completion), a sub-group analysis using chi-square tests was conducted to compare differences in exclusive breastfeeding rates among mothers with infected or suspected of infection during pregnancy, birth, or postpartum to determine the influence of the COVID-19 on early postpartum breastfeeding practices. Exclusive breastfeeding rates over time was also analyzed and tested using a Cochran–Armitage test for trend. All tests were two-sided and *p* ≤ 0.05 was considered statistically significant. Statistical analyses were conducted in Stata version 14 (Stata Corporation, College Station, TX, USA) and R version 4.1.1 (R Foundation for Statistical Computing, Vienna, Austria).

## Results

Among the 235 mothers who gave birth during the COVID-19 pandemic in Israel, 160 (68.1%) exclusively breastfed, 65 (27.7%) partially breastfed, and 10 (4.2%) did not breastfeed at discharge, for a combined total of 75 (31.9%) non-exclusively breastfeeding mothers. The intrapersonal, interpersonal, organizational, and community and society factors of the socio-ecological approach are presented based on exclusive breastfeeding status in Table 1.

**Table 1:** Characteristics of responders according to the breastfeeding status at discharge (*n* = 235)

	Exclusive breastfeeding n (%)	No exclusive breastfeeding n (%)	*p*-value
**Intrapersonal factors**			
***Sociodemographic factors***			
*Maternal age*			
18–30	53 (33.1)	26 (34.7)	0.816
31–35	67 (41.9)	34 (45.3)	0.618
36 or older	40 (25.0)	15 (20.0)	0.399
*Parity*			
Primiparous	45 (28.1)	39 (52.0)	0.001
Multiparous	115 (71.9)	36 (48.0)	0.001
*Maternal education*			
High school	12 (7.5)	9 (12.0)	0.260
University degree	80 (50.0)	40 (53.3)	0.634
Graduate degree (Master/Doctorate)	68 (42.5)	26 (34.7)	0.235
*Religion*			
Jews	155 (96.9)	71 (94.7)	0.411
Other	1 (0.6)	2 (2.7)	0.240
Missing	4 (2.5)	2 (2.7)	> 0.99
*Giving birth in same country as mother’s origin*			
Yes	133 (83.1)	60 (80.0)	0.689
No	27 (16.9)	15 (20.0)	0.689
***Prenatal and birth factors***			
*Difficulty attending prenatal care*			
Yes/Sometimes	69 (43.1)	26 (34.7)	0.276
No, never/Almost never	91 (56.9)	49 (65.3)	0.276
*Faced barriers to access prenatal care*			
Yes, always/Nearly always	12 (7.5)	4 (5.3)	0.782
Sometimes	46 (28.7)	23 (30.7)	0.764
No, never/Almost never	102 (63.7)	48 (64.0)	0.970
*Mode of birth*			
Vaginal birth (spontaneous and instrumental)	142 (88.8)	58 (77.3)	0.036
Cesarean birth	18 (11.2)	17 (22.7)	0.036
**Interpersonal factors**			
***Social support***			
*Adequate visiting hours for partner/relatives*			
Excellent/good	92 (57.5)	40 (53.3)	0.548
Sufficient	44 (27.5)	21 (28.0)	0.936
Insufficient/very bad	24 (15.0)	14 (18.7)	0.477
*Presence of companion of choice during hospitalization*			
Yes, always/Nearly always	125 (78.1)	59 (78.7)	0.925
Sometimes	28 (17.5)	12 (16.0)	0.775
No, never/Almost never	7 (4.4)	4 (5.3)	0.746
**Organizational factors**			
*Skin-to-skin contact in first hour*			
Yes	134 (83.8)	48 (64.0)	0.001
No	26 (16.2)	27 (36.0)	0.001
*Early breastfeeding in first hour*			
Yes	128 (80.0)	40 (53.3)	< 0.001
No	32 (20.0)	35 (46.7)	< 0.001
*Full rooming-in (including night)*			
Yes	123 (76.9)	39 (52.0)	< 0.001
No	37 (23.1)	36 (48.0)	< 0.001
*Adequate breastfeeding support*			
Yes	98 (61.3)	39 (52.0)	0.231
No	62 (38.8)	36 (48.0)	0.231
*HCP professionalism*			
Excellent/good	119 (74.4)	42 (56.0)	0.005
Sufficient	38 (23.8)	28 (37.3)	0.031
Insufficient/very bad	3 (1.9)	5 (6.7)	0.114
**Community and society factors**			
*Geographic region*			
Central Israel/Tel Aviv	54 (33.8)	28 (37.3)	0.591
Jerusalem	34 (21.2)	10 (13.3)	0.147
Haifa/Northern Israel	23 (14.4)	13 (17.3)	0.557
Southern Israel	45 (28.1)	22 (29.3)	0.848
Missing	4 (2.5)	2 (2.7)	> 0.99
*Timing: Variant outbreaks*			
*Alpha* March 2020–30 June 2021	115 (17.9)	53 (70.7)	0.962
*Delta* 1 July 2021–1 December 2021	32 (20.0)	16 (21.3)	0.975
*Omicron* 2 December 2021-July 2022	13 (8.1)	6 (8.0)	> 0.99
*Timing: 50% vaccination of population*			
March 2020-February 2021	86 (53.1)	36 (47.4)	0.495
March 2021-July 2022	74 (45.7)	39 (51.3)	0.495

*Abbreviation*: *HCP* health care provider

**Fig. 1:**
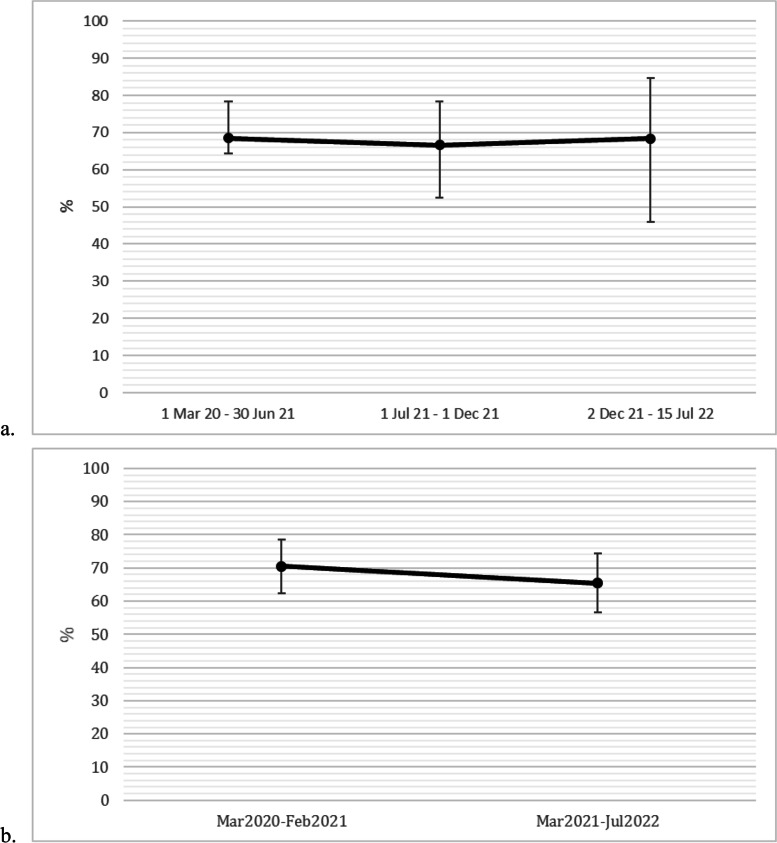
Trend analysis of exclusive breastfeeding rates over time in Israel (*n* = 235). Note: Noted points of time according to variant outbreaks (Alpha March 2020–30 June 2021; Delta 1 July 2021–1 December 2021); Omicron 2 December 2021-July 2022) (**a**) and according to 50% vaccination of the overall population (before and after March 2021) (**b**). No statistically significant effect of time was found (*p*-value = 0.902 and *p*-value = 0.411 for **a** and **b**, respectively)

Among the 152 (64.7%) respondents reporting COVID-19 infection status, 13.8% had been infected or suspected of infection during pregnancy, birth, or postpartum. Exclusive breastfeeding rates were not significantly different based on COVID-19 status (*p* = 0.884). Additionally, there was no significant change in the exclusive breastfeeding trend over time, based on outbreaks or vaccination rates (Fig. 1a and b).

Results of the multivariable logistic regression model demonstrate that factors significantly associated with exclusive breastfeeding among mothers in Israel were the intrapersonal factor of multiparity (adjusted OR 2.09; 95% Confidence Interval 1.01,4.35) and the organizational factors of early breastfeeding in the first hour (aOR 2.17; 95% CI 1.06,4.45), and rooming-in (aOR 2.68; 95% CI 1.41,5.07) (Table 2).

**Table 2:** Factors associated with exclusive breastfeeding, results of multivariable logistic regression (*n* = 235)

	**Adjusted OR (95% CI)**	***p*-value**
**Intrapersonal factors**		
*Parity*		
Primiparous	Reference category	
Multiparous	2.09 (1.01,4.35)	0.048
**Organizational factors**		
*Early breastfeeding in first hour*		
No	Reference category	
Yes	2.17 (1.06,4.45)	0.034
*Full rooming-in (including night)*		
No	Reference category	
Yes	2.68 (1.41,5.07)	0.002
*HCP professionalism*		
Excellent/good	Reference category	
Sufficient	0.55 (0.28,1.06)	0.073
Insufficient/poor	0.28 (0.06,1.33)	0.109

*Abbreviations*: *CI* confidence interval, *HCP* health care provider, *OR* odds ratio

*Note*: Reference categories were selected based on higher frequencies; OR are adjusted for maternal age, mode of birth, giving birth in same country as mother’s origin

Cox-Snell *R*^2^ = 0.150, Nagelkerke *R*^2^ = 0.210

## Discussion

Findings of the study highlight the importance of organizational factors in promoting exclusive breastfeeding in Israel. To the best of our knowledge, our study is the first published on exclusive breastfeeding outcomes at discharge. Similar to the larger study results on exclusive breastfeeding outcomes during the COVID-19 pandemic, early breastfeeding and rooming-in significantly influenced exclusive breastfeeding outcomes by discharge [13]. Even prior to the pandemic, these significant factors have been found to be positively associated with exclusive breastfeeding [25]. During the pandemic, organizational facilitators were challenged and professional breastfeeding assistance was reduced [11, 26]. In an online survey of lactation consultants working in hospitals and in community settings in Israel, over 78% felt skilled or very skilled in providing lactation support to breastfeeding mothers who were COVID-19 positive [27].

Many hospitals separated mothers and their infants without medical reason and restricted rooming-in despite being discouraged by evidence-based recommendations of the WHO [16] and researchers [14, 28]. It is noteworthy that among mothers in Spain who tested positive for COVID-19 infection in Baby-Friendly Hospital Initiative (BFHI) designated hospitals, skin-to-skin contact and rooming-in were implemented more frequently and exclusive breastfeeding rates were higher than non-designated hospitals (49% compared to 35%) [29], suggesting advantages of BFHI designation. While research of mothers from WHO European region found that rates of exclusive breastfeeding at discharge declined during the COVID-19 pandemic [13], mothers in Israel appear to have retained their breastfeeding patterns in relation to their intention [10] and timing in relation to outbreaks and vaccinations were not significant factors affecting exclusive breastfeeding rates.

Among the intrapersonal factors, only multiparity was associated with an increased likelihood of exclusive breastfeeding, as has been found by previous research conducted during the pandemic [13, 30]. The implication of this finding is that primiparous mothers and their infants would especially benefit from tailored breastfeeding guidance and support during pregnancy and while in the hospital, with particular attention to exclusive breastfeeding. An intervention study with a specific training for primiparous mothers throughout the perinatal period positively influenced exclusive breastfeeding rates [31], demonstrating the advantage of a focused intervention among new mothers. The remaining characteristics included in the current analysis were not significant in the multivariable model, including geographic region, which reflects consistency throughout the country. Different from many other studies that included the variable of insurance status, it is not a factor in Israel due to universal health insurance for citizens (98.7% of participants reported having national health insurance).

According to a primary Israeli healthcare organization’s breastfeeding recommendations during the pandemic, based on the WHO policy, healthy mothers should be encouraged to breastfeed their infants, while following basic hygiene precautions with infant care, breastfeeding, and pumping [32]. For breastfeeding mothers with suspected or confirmed COVID-19, as well as isolated mothers, the recommendations call for more careful behavior to prevent transmission of infection, such as frequent hand washing, cleaning surfaces, avoiding artificial nipple use, and thorough disinfection of pump parts [32]. The Israel MOH recommends that COVID-19 confirmed mothers should breastfeed while observing precautions including hand sanitizing, breast cleaning, mask wearing, and maintaining a 2-m distance when not breastfeeding or caring for the infant [33].

Limitations of the study are the small sample size, although we met the minimum required for the sample estimate. Additionally, this study was part of a large multi-country study that included 22 member countries of the WHO European Region and used an accepted dissemination plan of the online survey and established indicators of quality care measures selected from the WHO Standards [20]. Our sample included a highly educated group of mothers (91% with an academic education), similar to another recently conducted study in Israel [10], suggesting representativeness of online survey respondents among mothers in Israel. The study permitted voluntary reporting of COVID-19 status for reasons of perceived discomfort. Finally, some factors that might have affected exclusive breastfeeding at discharge, such as infant feeding difficulties, maternal mental health situation, access to lactation support services, infant sex, gestational age at birth, and BFHI hospital designation were not asked to women as the focus of the original study was maternal perception of quality of care. Having excluded multiple births and infants who were admitted to the NICU from the analysis, we likely included term infants thereby reducing potential confounding based on gestational age.

## Conclusions

This study points out the importance of implementing evidence-based breastfeeding recommendations during and after the pandemic, with particular attention to early breastfeeding during the hospital stay, and rooming-in. Early breastfeeding initiation and prevention of maternal-infant separation without a medical reason remain main contributors to exclusive breastfeeding at discharge, even during the COVID-19 pandemic. Primiparous mothers should be prioritized in receiving breastfeeding education and support throughout the perinatal period, particularly during the hospital stay. It is of crucial importance for the continuity of care for all mothers in order to ensure exclusive breastfeeding after discharge, as well as for monitoring breastfeeding data beyond the pandemic. Even during the pandemic, health care practitioners should implement and promote evidence-based breastfeeding recommendations to support exclusive breastfeeding.

## Data Availability

The datasets generated and analyzed for the current study may be available from the corresponding author, upon reasonable request.
